# Changes in Species Diversity Patterns and Spatial Heterogeneity during the Secondary Succession of Grassland Vegetation on the Loess Plateau, China

**DOI:** 10.3389/fpls.2017.01465

**Published:** 2017-08-29

**Authors:** Caili Sun, Zongzheng Chai, Guobin Liu, Sha Xue

**Affiliations:** ^1^State Key Laboratory of Soil Erosion and Dryland Farming on the Loess Plateau, Northwest A&F University Yangling, China; ^2^College of Forestry, Guizhou University Guiyang, China; ^3^Institute of Soil and Water Conservation, Chinese Academy of Sciences and Ministry of Water Resources Yangling, China

**Keywords:** abandoned fields, species composition, distribution pattern, soil erosion, power-law model

## Abstract

Analyzing the dynamic patterns of species diversity and spatial heterogeneity of vegetation in grasslands during secondary succession could help with the maintenance and management of these ecosystems. Here, we evaluated the influence of secondary succession on grassland plant diversity and spatial heterogeneity of abandoned croplands on the Loess Plateau (China) during four phases of recovery: 1–5, 5–10, 10–20, and 20–30 years. The species composition and dominance of the grassland vegetation changed markedly during secondary succession and formed a clear successional series, with the species assemblage dominated by *Artemisia capillaris*→ *Heteropappus* altaicus→ *A. sacrorum*. The diversity pattern was one of low–high–low, with diversity peaking in the 10–20 year phase, thus corresponding to a hump-backed model in which maximum diversity occurring at the intermediate stages. A spatially aggregated pattern prevailed throughout the entire period of grassland recovery; this was likely linked to the dispersal properties of herbaceous plants and to high habitat heterogeneity. We conclude that natural succession was conducive to the successful recovery of native vegetation. From a management perspective, native pioneer tree species should be introduced about 20 years after abandoning croplands to accelerate the natural succession of grassland vegetation.

## Introduction

Soil erosion is a severe environmental problem in China and around the world (Singh et al., 2006; Montgomery, 2007; Deng et al., 2012). Both governments and ecologists are paying more attention to the restoration of native vegetation, because this ecological solution can restore degraded land to prevent further erosion (Duncan and Chapman, 2003; Jiao et al., 2007; Chen et al., 2010). In 1999, the Chinese government launched its state-funded “Grain for Green Project” (GGP) to decrease erosion rates and to increase forest coverage by removing marginal cropland and land with steep slopes from agricultural production (Deng et al., 2012; Sun et al., 2016a). Much of the cropland on slopes >15° was abandoned and allowed to recover naturally without anthropogenic interventions. The GGP has since made a considerable contribution to the recovery of vegetation (Feng et al., 2013; Jiang et al., 2013). Inventory data indicate that about 14.67 × 10^6^ ha of cropland and 17.33 × 10^6^ ha of barren land were re-vegetated by semi-natural vegetation under the GGP during 1999–2010 (Chen et al., 2009).

The Loess Plateau of China has an area of approximately 62.4 × 10^4^ km^2^ and is well known for its long agricultural history and severe soil erosion. It was the main region targeted by the GGP program (Sun et al., 2016a). Here, land resources had been seriously depleted by the loss of soil and water, and the eco-environment of the Plateau badly degraded, which has directly affected local industrial and agricultural productivity (Shi and Shao, 2000). Nonetheless, strategies for conserving soil and water, including terracing, afforestation, natural rehabilitation, and the construction of check-dams, have been effective on the Plateau in the last several decades. The recovery and re-establishment of vegetation on abandoned cropland is one of the primary measures for soil erosion control on the Plateau (Wang et al., 2011; Zhao et al., 2013).

These abandoned fields were re-colonized naturally by the surrounding vegetation, which increased their vegetation cover with long-term natural recovery (Sun et al., 2016b). However, dramatic shifts in species abundance and composition can often occur during secondary succession (Chabrerie et al., 2003). Species-diversity patterns and spatial heterogeneity are important properties of plant communities and have been identified as indispensable parameters for evaluating the quality and sustainability of threatened and managed plant communities (Chen et al., 2007a; Guan et al., 2016). Species diversity data can provide information on the susceptibility to invasion, trophic structure, and ecosystem resilience (Nichols and Nichols, 2003), while spatial heterogeneity is the degree of aggregation and patchiness of co-occurring species in a community (Tsuki et al., 2005). Analyzing the developmental pathways of species-diversity patterns and spatial heterogeneity can provide valuable information on the main drivers of succession, especially when it is free of anthropogenic influence, as well as a solid theoretical basis for vegetation recovery and biodiversity conservation (Facelli et al., 1987).

Previous studies have demonstrated that secondary succession of grassland vegetation can effectively improve the physicochemical properties of soil (Sun et al., 2016b), and that vegetation cover can decrease soil erodibility (Wang et al., 2013) on the Loess Plateau. Soil organic carbon, clay, and total nitrogen contents and multifractal parameters have all increased substantially in the 35 years since cropland was first abandoned, whereas soil erodibility has gradually decreased to a steady stage after 28 years of restoration. Few comprehensive studies, however, have analyzed the variation in the composition and structure of grassland vegetation on the plateau during secondary succession after cropland abandonment.

In this study, we investigated how the duration of secondary succession influences the species-diversity pattern and spatial heterogeneity of grassland vegetation of abandoned cropland on the Loess Plateau by evaluating four recovery phases: 1–5, 5–10, 10–20, and 20–30 y. Specifically, we addressed two questions: (1) How do plant species composition, diversity patterns, and spatial heterogeneity change during secondary succession? and (2) What are the ecological implications for the natural recovery of this grassland vegetation?

## Materials and methods

### Study site

**Figure 1:**
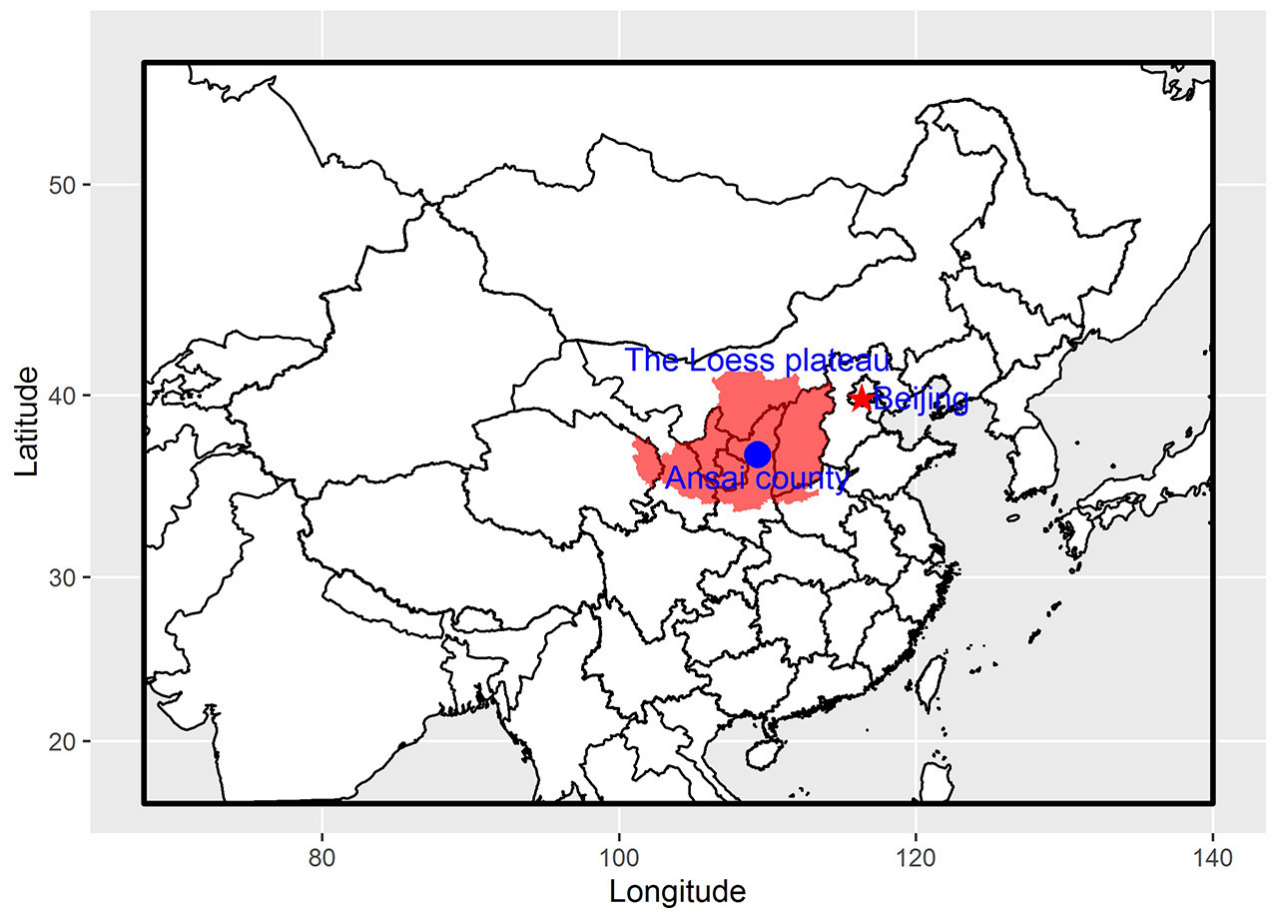
Location of the study site (Ansai County) on the Loess Plateau, China.

A field survey was conducted in the Ansai County of Shaanxi Province, China (Figure 1). Ansai County, located in the central part of the Loess Plateau, is well known for its extensive soil erosion. This area has a typical semiarid continental climate, with a mean annual temperature of 8.8°C and a mean annual precipitation of 549.1 mm. Precipitation varies seasonally, however, with 74.3% of it falling from June to September (Chen et al., 2007b; Sun et al., 2016a). Most native trees and shrubs were replaced by agricultural crops and were retained only at field edges and on gully slopes (Jiao et al., 2007). The current major land-use types are forest, shrubland, grassland, cropland, abandoned cropland, and residential areas in a mosaic rural landscape pattern (Jiao et al., 2012; Sun et al., 2016c). The typical vegetation currently found in the study area includes trees, such as *Populus davidiana* Dode., *Robinia pseudoacacia* Linn., and *Quercus wutaishanica* Blume; shrubs, such as *Hippophae rhamnoides* Linn. and *Caragana korshinskii* Kom.; and herbs, such as *Stipa bungeana* Trin. and *Artemisia sacrorum* Ledeb.

### Field sampling

We used the approach of substituting space for time—i.e., using plots of different ages in the same study area—to investigate grassland vegetation dynamics in the recovery phases during secondary succession. Data were collected in September 2011 from a total of 36 sampling plots (each 20 m × 20 m, “L-quadrats”) at nine locations and from four recovery phases after cropland abandonment: 1–5, 5–10, 10–20, and 20–30 y. Three 1 m × 1 m quadrats (108 “S-quadrats” in total) were chosen along the diagonals of each plot for detailed inventories of the grassland vegetation. All trees, shrubs, and herbs were identified and measured for their base diameter, layer coverage, total height, and growth condition. Local villagers and land documents verified the time of abandonment of each plot. Farming practices had been similar across all nine locations for >40 y before their abandonment. These abandoned croplands were allowed to undergo natural vegetation succession with no influence from human activity (Sun et al., 2016b).

### Data analysis

#### Analysis of beta diversity

Beta diversity measures the variation or differences in species composition among sites or communities, which can be due to species losses from site to site (nestedness) or to species replacement between sites (turnover) (Baselga and Orme, 2012; Cardoso et al., 2015). Baselga (2010, 2012) developed a unified framework for assessing beta diversity, in which one calculates the total dissimilarity using the Sørensen or Jaccard indices and their components of turnover and nestedness. Here, we used only the Sørensen index:

$$\begin{array}{lll} \beta_{\text{sor}}=\beta_{\text{sim}}+\beta_{\text{sne}} & \equiv & \frac{b+c}{2a+b+c}\\ = & \frac{b}{b+a}+\left(\frac{c-b}{2a+b+c}\right)\left(\frac{a}{b+a}\right) \end{array}$$

where β_sor_ is Sørensen dissimilarity, β_sim_ is Simpson dissimilarity (i.e., turnover component of Sørensen dissimilarity), β_sne_ is the nestedness component of the Sørensen dissimilarity, *a* is the number of species shared between two cells, *b* is the number of unique species at the poorest site, and *c* is the number of unique species at the richest site.

#### Rarefaction and extrapolation curves with hill numbers

Hill numbers—i.e., the effective numbers of species—are often used to characterize the taxonomic, phylogenetic, or functional diversity of a species assemblage (Chao et al., 2014a). This measure allows for the differential weighting of rare and abundant species, as the diversity indices collectively do, but its meaning is intuitively easier to understand (Hill, 1973). Integrated curves based on sampling theory, which smoothly link extrapolation and rarefaction, can standardize samples based on their size or completeness, and can also facilitate the comparison of biodiversity data. Chao et al. (2014b) applied a unified approach for both sample- and individual-based data for estimating rarefaction and extrapolation curves for the first three Hill numbers to characterize the diversity of a species assemblage: species richness (*q* = 0), the exponential of the Shannon entropy (Shannon diversity, *q* = 1), and the inverse Simpson concentration (Simpson diversity, *q* = 2). These proposed estimators are accurate for both rarefaction and short-range extrapolation.

We compared species-diversity patterns by using the rarefaction and extrapolation curves with the Hill numbers. Constructing the extrapolated and rarefied curves produced patterns based on data for incidence and abundance, respectively. Species diversity (species richness and Shannon and Simpson diversities) was estimated as the mean of 200 bootstrap replications, with 95% confidence intervals drawn (Chao et al., 2014a; Chai and Wang, 2016).

#### Power-law model

Shiyomi et al. (2001) proposed a power-law model for evaluating the amount of spatial heterogeneity for each plant species of a grassland community. The grassland vegetation was investigated by setting the *N* large quadrats (the “L-quadrats”), each of which was divided into *n* (*n* ≥ 2) small equal quadrats (the “S-quadrats”) (Shiyomi et al., 2001; Song et al., 2005; Tsuki et al., 2005). A random occurrence of individuals of species *i* in the *n* S-quadrats of an L-quadrat will be binomially distributed, with *p*_*i*_ representing the overall probability of occurrence of species *i*. The variance of the actual frequency of occurrence of species *i* is *v*_*i*_/*n*^2^, where *v*_*i*_ is the variance of observed counts for species *i* among the L-quadrats. The variance of occurrence is also given by *p*_*i*_(1-*p*_*i*_)/*n* if the occurrence of species *i* is also random. The relationship can be extended for the analysis of a community with *s* species as follows:

$$\begin{array}{l} \frac{v_{i}}{n^{2}}=\alpha^{\prime }{\left[\frac{p_{i}\left(1-p_{i}\right)}{n}\right]}^{\beta }\zeta_{i},i=1,2,\ldots ,s, \end{array}$$

where α′ and β are constants intrinsic to the plant community, and ζ is a random variable. For the logarithms of both variances, denoted by *y*_*i*_ = lg(*v*_*i*_*/n*^2^) and *x*_*i*_ = lg[*p*_*i*_(1-*p*_*i*_)/*n*], the relationship between *y*_*i*_ and *x*_*i*_ is expressed by the simpler regression:

$$\begin{array}{l} y_{i}=\alpha +\beta x_{i}+\varepsilon_{i},\text{i}=1,2,\ldots ,\text{s}, \end{array}$$

where α = lgα′and β are constants estimated from the samples, and ε_*i*_ = lgζ_*i*_ is the residual term for the difference of species *i* from the regression line, which indicates the community tendency. Species on the *y* = *x* line have random patterns, species above the line have aggregated patterns, and species below the line have regular patterns. The tendency of the spatial patterns in the community is random when α = 0 and β = 1; however, the distribution of individual species may not be random, depending on the value of ε_*i*_.

For regression lines above *y* = *x* over the range of *x*, the spatial patterns of a community tend to be more heterogeneous than those found in a random distribution. The degree of spatial heterogeneity of species *i* is expressed by δ_*i*_ as:

$$\begin{array}{l} \delta_{i}=\alpha +\left(\beta -1\right)x_{i}+\varepsilon_{i} \end{array}$$

The distribution of δ_*i*_ may thus be similar to that of ε_*i*_ if β is near 1 and α is near 0. The heterogeneity of the entire community (δ_*c*_) can be expressed by the weighted average heterogeneity:

$$\begin{array}{l} \delta_{c}=\overset{s}{\underset{i=1}{\sum }}p_{i}s_{i}/\overset{s}{\underset{i=1}{\sum }}p_{i} \end{array}$$

### Statistical analyses

All statistical analyses used the R version 3.3.2 (R Core Team, 2017). Beta diversity was analyzed using the betapart package (Baselga and Orme, 2012), and the rarefaction and extrapolation curves were compiled by using the iNEXT package (Hsieh et al., 2016). The figures were drawn and the data manipulated by using the ggplot2 (Hadley, 2007a) and reshape2 (Hadley, 2007b) packages, respectively.

## Results

### Species composition of the grassland vegetation

We identified a total of 64 plant species, belonging to 50 genera and 23 families, among the 5634 live plants found in the 108 quadrats spanning the four recovery phases on the Loess Plateau (see Table S1). The numbers of families, genera, and species and the species abundances increased gradually through the recovery phases of 1–5 to 10–20 y, but then they abruptly decreased to a low level after 20–30 y of natural secondary succession (Table 1).

**Table 1:** Summary of the attributes of the sampling plots for the grassland vegetation on abandoned croplands in the four recovery phases on the Loess Plateau, China.

**Item**	**Recovery phase (y)**				**Total**
	**1–5**	**5–10**	**10–20**	**20–30**	
Site number	9	9	9	9	36
Sample number	27	27	27	27	108
Sample area (m^2^)	27	27	27	27	108
Family number	9	14	17	14	23
Genera number	26	33	37	31	50
Species number	31	37	47	39	64
Species abundance	1,246	1,398	1,693	1,297	5,634

The species *A. capillaris* Thunb., *Sonchus oleraceus* Linn., *S. bungeana* Trin., and *Bothriochloa ischaemum* (Linn.) Keng had the highest IVs and were the dominant species in the 1–5y recovery phase; *H. altaicus* (Willd.) Novopokr., *B. ischaemum, Setaria viridis* (Linn.) Beauv., and *Leymus secalinus* (Georgi) Tzvel. were the dominant species at 5–10 y; *A. sacrorum, A. leucophylla* (Turcz. ex Bess.) C. B. Clarke, *S. bungeana*, and *H. altaicus* were the dominant species at 10–20 y; and *A. sacrorum, A. leucophylla, Lespedeza bicolor* Turcz., and *S. bungeana* were the dominant species at 20–30 y. The grassland at the site underwent a clear successional series, with species assemblages dominated by *A. capillaris*→ *H. altaicus*→ *A. sacrorum*.

**Figure 2:**
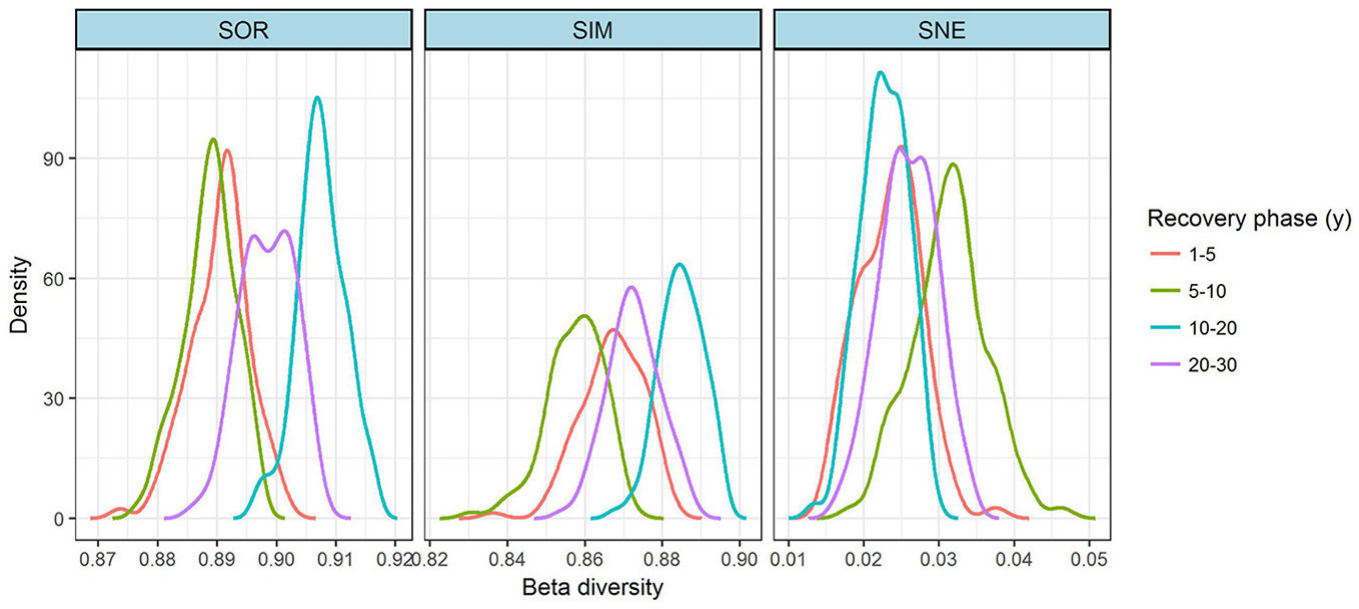
Density plots for the dissimilarity distribution of species composition, based on a beta-diversity analysis for the grassland vegetation on abandoned croplands among the four recovery phases on the Loess Plateau, China. The partition of total dissimilarity (SOR) into turnover dissimilarity (SIM) and nestedness (SNE) components. Density estimation was performed by using the kernel smoothing method.

The variation in species composition among the four recovery phases was estimated by the beta-diversity analysis (Figure 2). Total dissimilarity (β_*SOR*_) and turnover dissimilarity (β_*SIM*_) showed similar trends among the four phases: being highest for 10–20 y, but with similar values at 5–10 y and 20–30 y, but lowest for 5–10 y. The nestedness dissimilarity (β_*SNE*_) clearly differed among the four recovery phases in this order: 10–20 > 20–30 > 5–10 > 1–5 y.

### Diversity patterns of the grassland vegetation

#### Comparison of species diversity based on the abundance data

**Figure 3:**
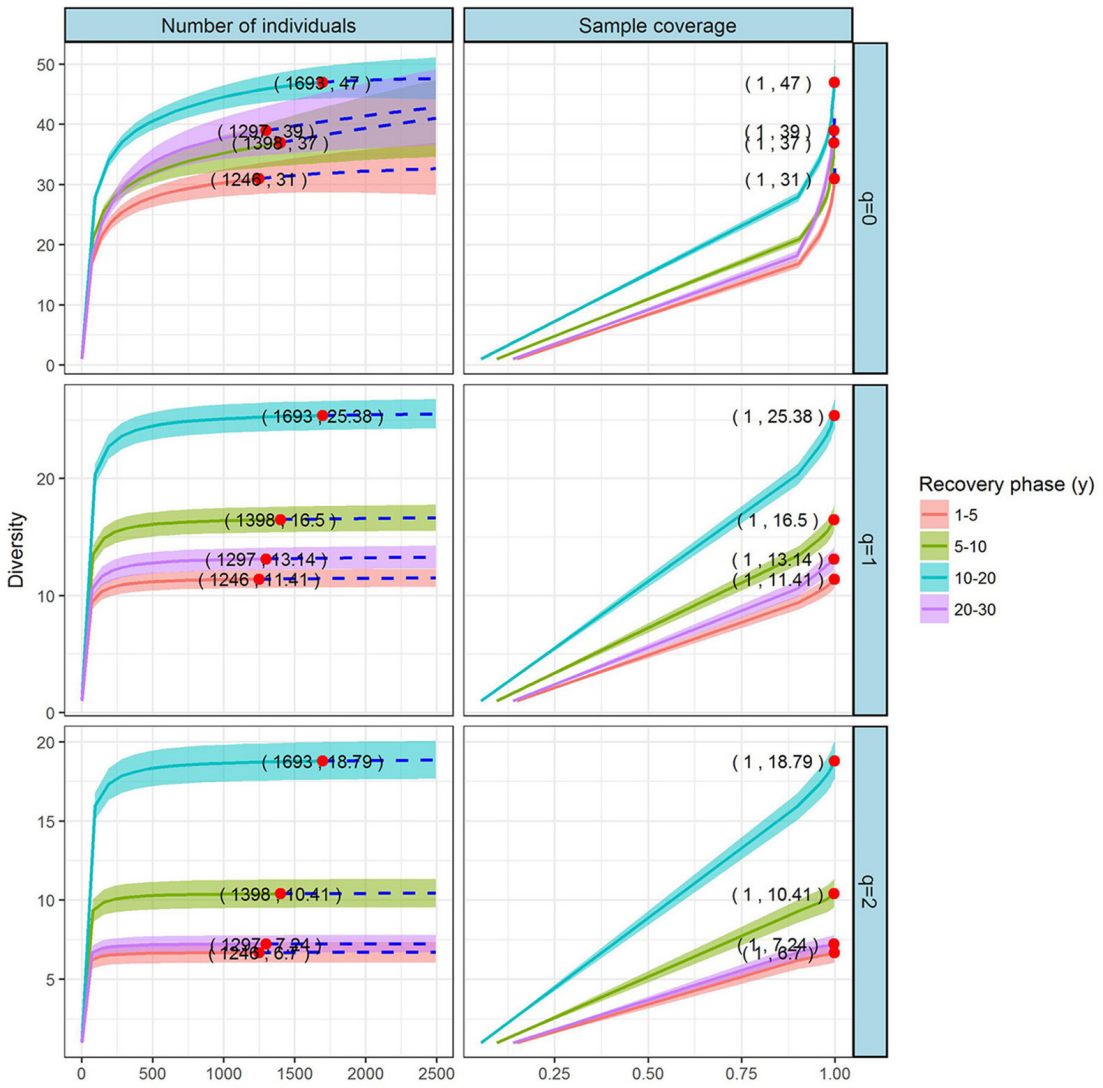
Individual- and coverage-based rarefaction and extrapolation curves, based on the Hill numbers (*q* = 0, 1, 2) for the grassland vegetation on abandoned croplands among the four recovery phases on the Loess Plateau, China. The 95% confidence intervals (shaded regions) were obtained by bootstrapping (200 replications). Reference samples are denoted by solid dots; the numbers in parentheses are the respective sample size and observed Hill number of each reference sample. In this study, we used the first three Hill numbers to characterize the diversity of a species assemblage based on the individual-based data: species richness (*q* = 0), the exponential of the Shannon entropy (Shannon diversity, *q* = 1), and the inverse Simpson concentration (Simpson diversity, *q* = 2). The proposed estimators are accurate for both rarefaction (solid line) and short-range extrapolation (dashed line, up to double the reference sample size).

Coverage- and individual-based rarefaction and extrapolation curves for the Hill numbers *q* = 0, 1, and 2 compared the plant diversities among the four recovery phases (Figure 3). The reference sample sizes (i.e., number of individual plants) were 1,246, 1,398, 1,693, and 1,297 for the phases of 1–5, 5–10, 10–20, and 20–30 y, respectively. The base coverage was 1.0, indicating that the sample size was nearly complete for these recovery phases. We extrapolated the reference sample size to 2,492. The corresponding observed species richness (*q* = 0), Shannon diversity (*q* = 1), and Simpson diversity (*q* = 2) showed consistent trends: increasing gradually from 1–5 y to 10–20 y, yet remaining stable with diversity peaking at 10–20 y, then decreasing in the final phase (20–30 y). Nonetheless, the confidence intervals for species richness overlapped.

#### Comparison of species diversity based on the incidence data

**Figure 4:**
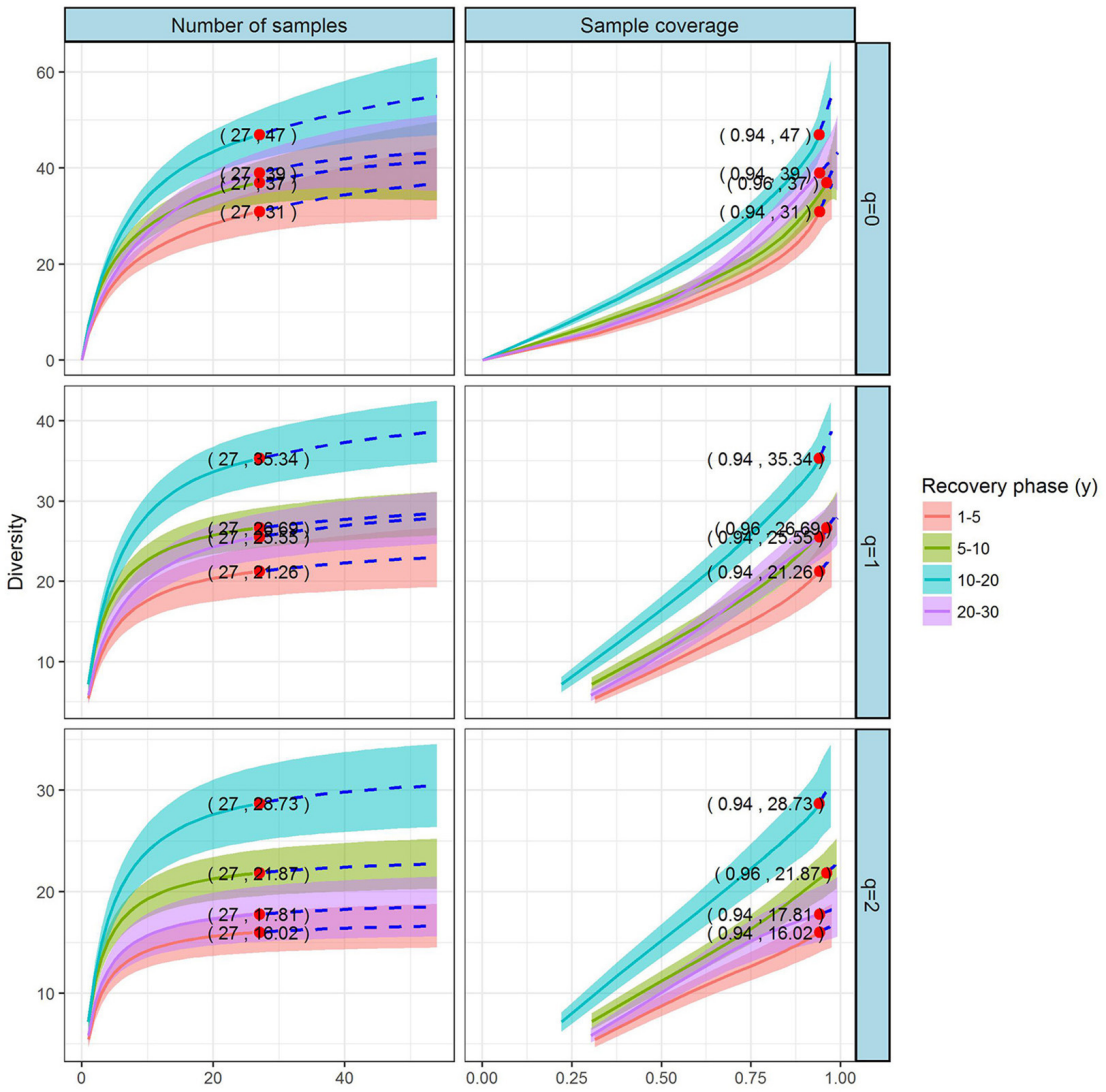
Sample- and coverage-based rarefaction and extrapolation curves based on the Hill numbers (*q* = 0, 1, 2) for the grassland vegetation on abandoned croplands among the four recovery phases on the Loess Plateau, China. The 95% confidence intervals (shaded regions) were obtained by bootstrapping (200 replications). Reference samples are denoted by solid dots, and the numbers in parentheses are the respective sample size and observed Hill number for each reference sample. In this study, we used first three Hill numbers to characterize the diversity of a species assemblage based on the sample-based data: species richness (*q* = 0), the exponential of the Shannon entropy (Shannon diversity, *q* = 1), and the inverse Simpson concentration (Simpson diversity, *q* = 2). The proposed estimators are accurate for both rarefaction (solid line) and short-range extrapolation (dashed line, up to double the reference sample size).

Coverage- and sample-based rarefaction and extrapolation curves for Hill numbers *q* = 0, 1, and 2 for compared the plant diversities among the four recovery phases (Figure 4). All four phases had the same reference sample size of 27. The base coverage was ≥ 0.94, close to 1.0, suggesting adequate sample sizes that could satisfy the research requirements. We extrapolated the reference sample size to 54. The corresponding observed species richness (*q* = 0), Shannon diversity (*q* = 1), and Simpson diversity (*q* = 2) had patterns consistent with those for species diversity based on the abundance data (although some of the confidence intervals for species diversity overlapped).

In sum, a comparison of species diversity based on both abundance data and incidence data found that the diversity pattern of grassland vegetation followed a trend of “low–high–low,” with species diversity highest during 10–20 y of succession.

### Spatial heterogeneity of the grassland vegetation

**Figure 5:**
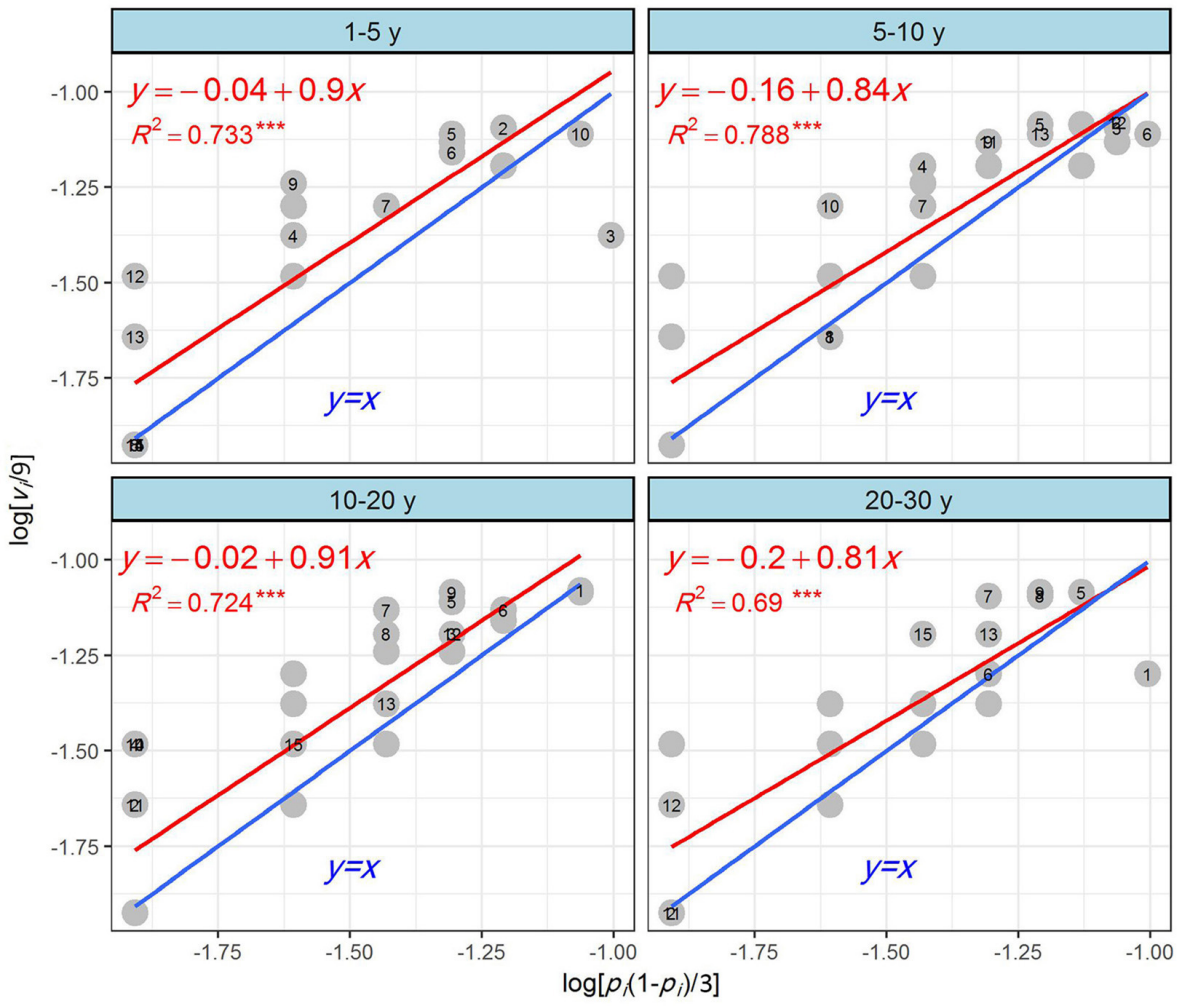
Application of the power law to the grassland plant species on abandoned croplands among the four recovery phases on the Loess Plateau, China. A random occurrence of individuals of species *i* in *n* (*n* = 3 in this study) S-quadrats within a L-quadrat will be binomially distributed, with *p*_*i*_ representing the overall probability of occurrence of species *i*. The variance of the actual frequency of occurrence of species *i* is *v*_*i*_/*n*^2^, where *v*_*i*_ is the variance of observed counts for species *i* among the L-quadrats. The variance of occurrence is also given by *p*_*i*_(1-*p*_*i*_)/n, when the occurrence of species *i* is random. For logarithms of both variances, denoted by *y*_*i*_ = lg(*v*_*i*_*/n*^2^) and *x*_*i*_ = lg[*p*_*i*_(1-*p*_*i*_)/*n*], the relationship between *y*_*i*_ and *x*_*i*_ is expressed by simpler linear regression. Species *i* forms a random pattern if (*x*_*i*_, *y*_*i*_) is on the *y* = *x* line; species *i* forms a more heterogeneous pattern if the coordinates of species *i* are above the *y* = *x* line. The farther above the line, the more heterogeneous is the pattern. The regression line indicates the tendency of species in the entire community. The red line is the regression line and the blue line is the *y* = *x* line. The dominant species, H1–H15, were as follows: H1, *Artemisia sacrorum* Ledeb.; H2, *Setaria viridis* (Linn.) Beauv.; H3, *A. capillaris* Thunb.; H4, *Bidens pilosa* Linn.; H5, *Stipa bungeana* Trin.; H6, *Heteropappus altaicus* (Willd.) Novopokr.; H7, *Lespedeza daurica* (Laxm.) Schindl.; H8, *A. leucophylla* (Turcz. ex Bess.) C. B. Clarke; H9, *L. bicolor* Turcz.; H10, *Sonchus oleraceus* Linn.; H11, *Leymus secalinus* (Georgi) Tzvel.; H12, *Dracocephalum moldavica* Linn.; H13, *Potentilla bifurca* Linn.; H14, *Roegneria kamoji* Ohwi; and H15, *A. argyi* Levl. et Vant.

Figure 5 shows a graphic representation of the power law for the four recovery phases. The high *R*^2^ values (coefficients of determination) for all phases indicated that the power law adequately explained the mapping data and successfully identified high spatial heterogeneity. All of the regression lines were mostly above the *y* = *x* line over the observed ranges of *x*. The spatial patterns of the four phases were judged to be more heterogeneous than would be expected from a random pattern, as they displayed an overall aggregated pattern according to the power-law graph.

**Figure 6:**
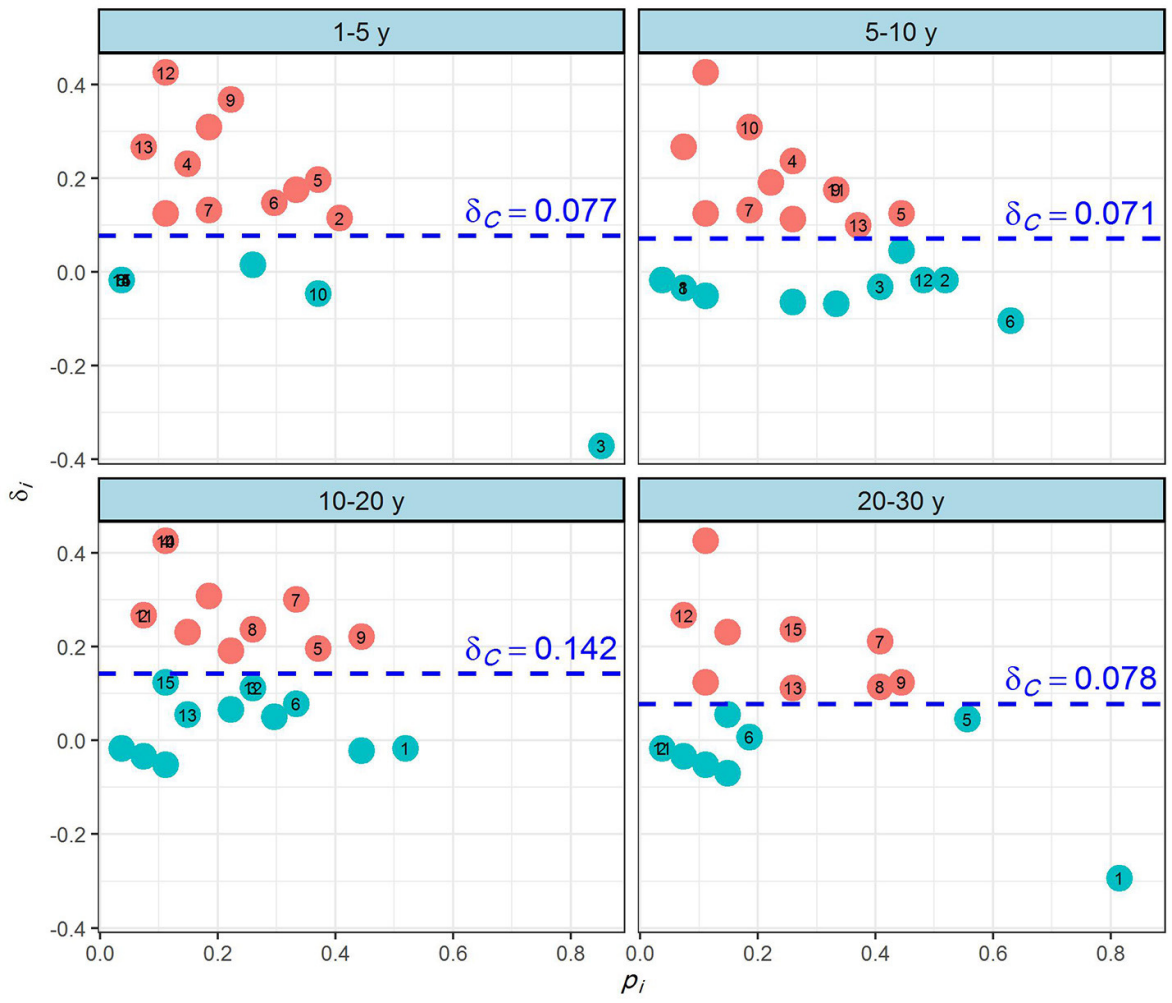
Relationship between occurrence (*p*_*i*_) and spatial heterogeneity (δ_*i*_) of the grassland plant species on abandoned croplands among the four recovery phases on the Loess Plateau, China. The *p*_*i*_ is the proportion of occurrence for species *i*, the δ_*i*_ is the degree of spatial heterogeneity of species *i*, and the δ_*c*_ is the heterogeneity of the entire community. Generally, a large δ_*i*_ indicates that the species are aggregated in large clumps, and a small δ_*i*_ indicates that the species are randomly distributed; the δ_*c*_ line indicates the spatial heterogeneity for the entire community. Refer to Figure 5 for the species names (H1–H15).

Figure 6 shows the relationship between the occurrence, *p*, and spatial heterogeneity, δ, of the four recovery phases. Here, we used the 15 most dominant species with their maximum abundances (see Table S1) as examples to illustrate their spatial heterogeneity in the four recovery phases.

In the first 1–5 y of succession, *Dracocephalum moldavica* Linn. (H12), *L. bicolor* (H9), *Potentilla bifurca* Linn. (H13), and *Bidens pilosa* Linn. (H4) were highly heterogeneous. By contrast, *L. daurica* (Laxm.) Schindl. (H7), *S. viridis* (H2), *A. argyi* Levl. et Vant. (H15), and *A. leucophylla* (H8) were less heterogeneous and had intermediate δ values. The species *A. capillaris* Thunb. (H3) had an approximately random pattern. In the next phase, of 5–10 y, *S. oleraceus* (H10), *B. pilosa* (H4), *L. secalinus* (H11), and *L. bicolor* (H9) were all highly heterogeneous, whereas *S. bungeana* (H5), *P. bifurca* (H13), *D. moldavica* (H12), and *S. viridis* (H2) were less heterogeneous and had intermediate δ values. The distribution of species *H. altaicus* (H6) followed an approximately random pattern. In the third, of 10–20 y, *S. oleraceus* (H10), *B. pilosa* (H4), *L. secalinus* (H11), and *S. viridis* (H2) were all highly heterogeneous. Conversely, *L. bicolor* (H9), *S. bungeana* (H5), *A. argyi* (H15), and *D. moldavica* (H12) were less heterogeneous and had intermediate δ values. The species *A. sacrorum* (H1) had an approximately random pattern. In the final phase assessed, that of 20–30 y, *D. moldavica* (H12), *A. argyi* (H15), and *L. daurica* (H7) were highly heterogeneous. *P. bifurca* (H13), *A. leucophylla* (H8), *S. bungeana* (H5), and *H. altaicus* (H6) were less heterogeneous and had intermediate δ values, while *A. sacrorum* (H1) showed a more or less random pattern.

The spatial heterogeneity for the entire grassland community (δ_*c*_) increased gradually for the recovery phases of 1–5 y (δ_*c*_ = 0.077), 5–10 y (δ_*c*_ = 0.071), and 10–20 y (δ_*c*_ = 0.142), was largest in the 10–20 y phase, but then abruptly decreased to a low level in the phase of 20–30 y (δ_*c*_ = 0.078).

## Discussion

### Changes in species composition during secondary succession of the grassland vegetation

After cropland abandonment, the grassland species composition and dominance changed markedly during the four phases of 1–30 y of secondary succession. A clear successional series emerged, whereby the species assemblage was dominated by *A. capillaris*→ *H. altaicus*→ *A. sacrorum*. This same successional series has been reported by multiple studies on the Loess Plateau (Zou et al., 1998; Wang et al., 2011; Sun et al., 2016b), which have generally found that the annual herbaceous species, *A. capillaris* (a common pioneer species), was often later succeeded by the perennial species *A. sacrorum* during grassland succession. The perennial species *H. altaicus* has always been an associated species, and though it continues to survive and grow over the long term it cannot ultimately form a climax grassland community. The importance value of *A. sacrorum* increased across the recovery phases; it was the dominant species in the 10–20 y phase, but obviously more so at 20–30 y. We therefore conclude that the grassland vegetation at our study site is adaptable to the environment: it was able to form a stable community after 20 years of natural recovery of abandoned cropland.

### Changes in diversity patterns during secondary succession of the grassland vegetation

The diversity pattern of the grassland vegetation had a trend of low–high–low, with diversity greatest in the 10–20 y phase. This pattern corresponds to a hump-backed model with maximum diversity at intermediate stages (Grime, 1973). Species diversity in this model is low during the early stages of succession, which are colonized by a limited number of pioneer plants. Other species invade the area as succession progresses, so that many mid- and late-successional species compose the intermediate stages, decreasing species diversity, whereas the late stage becomes dominated by a few late arrivals (Begon et al., 2006; Madeira et al., 2009). Our results also showed that both the pioneer species (*A. capillaris, S. oleraceus*, and *B. pilosa*) and the late-successional species (*A. sacrorum, S. grandis* P. Smirn., and *Parinia heterophylla* Bunge) coexisted in the 10–20 y phase. Chen et al. (2014) also found this pattern and noted that community coverage, plant density, diversity, and biomass all gradually increased during the secondary succession of grassland vegetation, with the maximum levels reached within about 20 years. We therefore conclude that the Loess Plateau grassland had attained a stable plant community through 30 years of invasion, competition, diffusion, and ecesis.

### Changes in spatial heterogeneity during secondary succession of the grassland vegetation

An aggregated pattern was prevalent in the four recovery phases of the grassland, consistent with multiple findings in arid or semiarid natural grasslands in China and elsewhere (Song et al., 2005; Tsuki et al., 2005; Chen et al., 2007a; Guan et al., 2016). Condit et al. (2000) noted that wind-dispersed species were not as well dispersed as the animal-dispersed species and tended to have aggregated distributions. The majority of herbaceous plants found in our study area produce wind-dispersed seeds, and some, such as the perennial species *H. altaicus* and *A. vestita*, can propagate by root shoots, which can further contribute to a highly aggregated spatial pattern. The ecological environments in arid and semiarid areas, especially on the Loess Plateau, are harsh and complex, the habitats are highly heterogeneous, both soil moisture and fertility are unevenly distributed, and the grazing disturbance is high—this great environmental heterogeneity determines the variation and aggregated distribution of the vegetation. Jiao et al. (2007) reported that the time of abandonment, total P content, and soil-water content were the key factors affecting the establishment of vegetation on the Loess Plateau. Many studies have emphasized that the availability of soil water to plants is a critical factor controlling productivity and heterogeneity of plant distributions, and of other life forms, in arid and semiarid areas (e.g., Snyman, 2002; Noymeir, 2003; Diaz et al., 2004; Chen et al., 2010). Liu et al. (2014) and Song et al. (2005) suggested that topography was another heterogeneous factor that could affect the structure and dynamics of vegetation communities, because topography also controls soil moisture that drives plant distributional pattern and diversity. This view was supported by the findings by Moeslund et al. (2013). The pronounced aggregated pattern reported by us can thus be linked to the dispersal properties of herbaceous plants and the higher environmental heterogeneity on the Loess plateau.

### Ecological implications

Natural succession assists in the recovery of vegetation. However, natural succession over a long period (>20 years) may be needed to establish stable vegetation cover, especially in grazed areas or where erosion is still a large problem. Our results indicated that successional age was a key factor in controlling species composition and diversity, as it further affected the recovery and development of the vegetation. Furthermore, the succession of grassland vegetation may be accelerated by introducing pioneer tree species. The succession of vegetation on the Plateau is as follows: annual herbs→ perennial herbs→ shrubs→ pioneer trees→ climax trees. Our study found that the grassland communities which formed after 20 years of natural recovery on abandoned cropland were dominated by some shrub and semi-shrub species, namely *A. sacrorum, A. leucophylla, L. bicolor*, and *L. daurica*. We conclude that the grassland vegetation in our study was on a positive and progressive succession trajectory, such that we predict the plant community at our site will eventually transition to a stable and climax tree community (if given the time). We therefore recommend that some native pioneer tree species, such as *Pinus tabuliformis* Carr., *Betula platyphylla* Suk., and *P. davidiana* Dode., be considered for introduction to the grassland 20 years after abandonment, to accelerate the process of succession and to promote community stability.

## Conclusions

The species composition, diversity patterns, and spatial heterogeneity changed markedly during the secondary succession (1–30 y) of grassland vegetation on the Loess Plateau. The pattern of plant diversity was of low, then high, and then low again, with the highest diversity observed in the 10–20 y phase. An aggregated spatial pattern was pronounced throughout the entire period of grassland recovery. Natural succession assisted the recovery of vegetation to abandoned croplands. However, the results suggest that 20 years after abandonment occurs, native pioneer tree species should be introduced to accelerate the natural succession of grassland vegetation.

## Author contributions

CS, SX, and GL initiated and designed the research. SX and GL obtained funding for this study. CS, SX, and GL collected the materials and performed the experiments. CS, ZC, and SX analyzed the data and wrote the manuscript. All authors read and approved the manuscript.

### Conflict of interest statement

The authors declare that the research was conducted in the absence of any commercial or financial relationships that could be construed as a potential conflict of interest.
