# Supplementary material for: Changes in Species Diversity Patterns and Spatial Heterogeneity during the Secondary Succession of Grassland Vegetation on the Loess Plateau, China
Source: Front Plant Sci. 2017 Aug 29;8:1465. doi: 10.3389/fpls.2017.01465 (PMC5581942; doi:10.3389/fpls.2017.01465)
Supplement: Supplementary file 1 [file Table1.PDF]

Table S1 Species composition and importance value (IV) index for grassland plant species of abandoned farmland in the different recovery phase in the Loess Plateau, China

ID	Chinese name	Scientific name	Family name	Abundance	Importance value (%) in the different recovery phase (a)			
					1~5	5~10	10~20	20~30
H1	铁杆蒿	*Artemisia vestita* Wall. ex Bess.	Compositae	508	—	1.42	11.62	33.99
H2	狗尾草	*Setaria viridis* (Linn.) Beauv.	Gramineae	468	7.27	8.96	0.45	0.66
H3	茵陈蒿	*Artemisia capillaris* Thunb.	Compositae	445	28.67	6.47	2.17	—
H4	鬼针草	*Bidens pilosa* Linn.	Compositae	420	1.6	6.1	1.35	—
H5	长芒草	*Stipa bungeana* Trin.	Gramineae	412	9.5	6.66	7.83	7.43
H6	阿尔泰狗娃花	*Heteropappus altaicus* (Willd.) Novopokr.	Compositae	375	4.46	11.1	6.7	3.56
H7	兴安胡枝子	*Lespedeza daurica* (Laxm.) Schindl.	Leguminosae	328	1.96	2.64	5.08	5.64
H8	茭蒿	*Artemisia leucophylla* (Turcz. ex Bess.) C. B. Clarke	Compositae	251	0.15	0.45	8.4	11.74
H9	胡枝子	*Lespedeza bicolor* Turcz.	Leguminosae	194	2.75	3.08	5.7	7.48
H10	苦苣菜	*Sonchus oleraceus* Linn.	Compositae	183	9.55	0.83	1.04	—
H11	赖草	*Leymus secalinus* (Georgi) Tzvel.	Gramineae	180	0.25	6.75	1.59	0.26
H12	香青兰	*Dracocephalum moldavica* Linn.	Labiatae	179	0.76	5.85	2.59	0.45
H13	二裂委陵菜	*Potentilla bifurca* Linn.	Rosaceae	147	0.3	4.89	2.04	2.75
H14	鹅观草	*Roegneria kamoji* Ohwi	Gramineae	137	0.25	—	4.71	—
H15	艾蒿	*Artemisia argyi* Levl. Et Vant .	Compositae	124	0.57	—	1.93	4.56
H16	豌豆	*Pisum sativum* Linn.	Leguminosae	102	1.74	1.33	1.89	0.66
H17	白羊草	*Bothriochloa ischcemum* (Linn.) Keng	Gramineae	94	8.29	10.59	3.94	1.25
H18	糙隐子草	*Cleistogenes squarrosa* (Trin.) Keng	Gramineae	76	1.31	2.45	6.38	2.57

H19	刺儿菜	*Cirsium setosum* (Willd.) MB.	Compositae	74	3.08	4.01	0.98	0.71
H20	地锦	*Parthenocissus tricuspidata* (Sieb. et Zucc.) Planch.	Vitaceae	74	3.54	1.71	0.1	0.12
H21	硬质早熟禾	*Poa sphondylodes* Trin.	Gramineae	59	1.48	1.31	0.42	—
H22	老鹳草	*Geranium wilfordii* Maxim.	Geraniaceae	55	4.55	0.72	1.46	0.84
H23	芦苇	*Phragmites australis* (Cav.) Trin. ex Steud.	Gramineae	55	—	1.2	1.8	0.38
H24	猪毛菜	*Salsola collina* Pall.	Chenopodiaceae	53	—	3.21	0.72	0.87
H25	异叶败酱	*Parinia heterophylla* Bunge	Valerianaceae	52	—	—	0.19	2.03
H26	大针茅	*Stipa grandis* P. Smirn.	Gramineae	49	—	—	0.4	1.8
H27	二色棘豆	*Oxytropis bicolor* Bunge	Leguminosae	47	0.68	—	3.81	0.2
H28	野百合	*Lilium brownii* F. E. Brown ex Miellez	Liliaceae	46	—	0.59	—	—
H29	菊叶委陵菜	*Potentilla tanacetifolia* Willd. ex Schlecht.	Rosaceae	45	2.09	—	2.44	0.43
H30	野菊花	*Dendranthema indicum* (Linn.) Des Moul.	Compositae	42	—	1.55	—	—
H31	披针苔草	*Carex lancifolia* C. B. Clarke	Cyperaceae	41	—	—	1.46	1.59
H32	甘菊	*Dendranthema lavandulifolium* (Fisch. ex Trautv.) Ling et Shih	Compositae	32	1.6	—	—	—
H33	苦荬菜	*Ixeris polycephala* Cass.	Compositae	28	0.79	0.87	1.07	1.38
H34	甘青针茅	*Stipa przewalskyi* Roshev.	Gramineae	28	—	—	2.5	—
H35	香青	*Anaphalis sinica* Hance	Compositae	26	1.27	1.69	—	0.17
H36	紫花地丁	*Viola philippica* Cav.	Violaceae	23	0.34	—	1	—
H37	早熟禾	*Poa annua* Linn.	Gramineae	21	0.67	—	0.92	0.3
H38	蒲公英	*Taraxacum mongolicum* Hand.-Mazz.	Compositae	21	0.21	0.94	1.38	—
H39	远志	*Polygala tenuifolia* Willd.	Polygalaceae	18	—	0.12	0.74	0.62
H40	披针叶黄华	*Thermopsis lanceolala* R.Br.	Leguminosae	17	—	—	0.66	0.43

H41	北京隐子草	*Cleistogenes hancei* Keng	Gramineae	16	—	—	0.17	0.78
H42	野棉花	*Anemone vitifolia* Buch.-Ham.	Ranunculaceae	15	—	0.43	—	1.67
H43	大果琉璃草	*Cynoglossum divaricatum* Stephan ex Lehmann	Boraginaceae	13	—	—	0.24	—
H44	佛子茅	*Calamagrostis epigeios* (L.) Roth	Gramineae	10	—	—	—	0.45
H45	草木犀状黄芪	*Astragalus melilotoides* Pall. var. tenuis Ledeb.	Leguminosae	8	—	—	0.17	0.86
H46	杠柳	*Periploca sepium* Bunge	Asclepiadaceae	7	—	0.53	0.47	—
H47	甘草	*Glycyrrhiza uralensis* Fisch.	Leguminosae	5	—	—	0.27	0.32
H48	沙打旺	*Astragalus adsurgens* Pall.	Leguminosae	4	—	0.09	—	0.22
H49	地构叶	*Speranskia tuberculata* (Bunge) Baill.	Euphorbiaceae	4	—	0.47	—	—
H50	枸杞	*Lycium chinense* Miller	Solanaceae	3	—	—	—	0.24
H51	野苜蓿	*Medicago falcata* Linn.	Leguminosae	3	0.18	0.26	—	—
H52	灰棘豆	*Oxytropis cana* Bunge	Leguminosae	2	—	—	0.16	—
H53	风毛菊	*Saussurea petrovii* Lipsch.	Compositae	2	—	0.23	0.19	—
H54	阴行草	*Siphonostegia chinensis* Benth.	Scrophulariaceae	2	—	—	0.44	—
H55	苦苣菜	*sonchus oleraceus* L.	Compositae	2	—	—	0.14	—
H56	黄花蒿	*Artemisia annua* Linn.	Compositae	1	—	—	—	0.15
H57	铁线莲	*Clematis florida* Thunb.	Ranunculaceae	1	—	0.11	—	—
H58	鹤虱	*Lappula myosotis* Moench	Boraginaceae	1	0.16	—	—	—
H59	野亚麻	*Linum stelleroides* Planch.	Linaceae	1	—	—	0.15	—
H60	茜草	*Rubia cordifolia* Linn.	Rubiaceae	1	—	—	0.17	—
H61	裂叶风毛菊	*Saussurea laciniata* Ledeb.	Compositae	1	—	0.2	—	—
H62	丁香	*Syzygium aromaticum* (L.) Merr．Et Perry	Myrtaceae	1	—	—	—	0.19

H63	唐松草	*Thalictrum aquilegifolium* Linn. var. sibiricum Regel et Tiling	Ranunculaceae	1	—	—	—	0.24
H64	榆树	*Ulmus pumila* Linn.	Ulmaceae	1	—	0.18	—	—
